# The effect of exercise on the prevention of gestational hypertension in obese and overweight pregnant women: An updated meta-analysis

**DOI:** 10.3389/fpubh.2022.923161

**Published:** 2022-08-15

**Authors:** Enli Xie, Huimin Tao, Mengqing Liu, Changchun Li, Qi Zhao

**Affiliations:** ^1^Department of Sports Training, Nanjing Sport Institute, Nanjing, China; ^2^Department of Chaohu Clinical Medicine, Anhui Medical University, Hefei, China; ^3^School of Physical Education, Spots Institute of Jingdezhen University, Jingdezhen, China

**Keywords:** physical exercise, gestational hypertension, meta-analysis, pregnancy, obese

## Abstract

**Background:**

Gestational hypertension (GH) is a common disease that seriously threatens the safety and health of pregnant women and their newborns. Physical exercise (PE) is widely recognized as a health maintenance method and it has numerous benefits. Studies on the association between PE and the risk of GH in obese and overweight pregnant women have generated controversial findings. This updated meta-analysis was performed to reassess the effects of PE on GH.

**Methods:**

The articles from inception to April 2022, presenting studies investigating exercise intervention and pregnancy outcomes were explored across several online databases. Heterogeneity among the included studies was estimated and tested by Q test and *I*^2^ statistic. Risk ratios (RRs) and 95% confidence intervals (CI) were calculated through either random-effect or fixed-effect models. Subgroup analyses, sensitivity analyses, and publication bias diagnoses were also conducted.

**Results:**

Twelve with 1,649 subjects were included. PE was associated with a reduced risk of GH in obese and overweight pregnant women (Pooled RR = 0.58, 95% CI = 0.42–0.81, *P* = 0.001; *I*^2^ = 24.3%). Subgroup analysis found significant trends amongst Eastern countries (RR = 0.59, 95% CI = 0.36–0.96, *P* = *0.033*). Sensitivity analysis suggested the results were stable. No publication bias was detected based on Begg's test and Egger's test.

**Conclusion:**

PE was associated with reduced risk of GH in obese and overweight pregnant women, especially in Eastern countries. More well-designed studies are still needed to further elaborate on these associations.

**Systematic review registration:**

CRD42022326183.

## Introduction

Over the past few decades, the number of overweight and obese people has been increasing, especially among women of reproductive age (1–3). Approximately 25% of women are overweight after childbirth and 20% of women are obese before pregnancy worldwide (4). However, it is reported that maternal obesity is associated with an increased risk of adverse pregnancy complications for both mother and baby (5–7). Studies have shown that children born to obese pregnant women might have a comparatively greater risk of suffering from cardiovascular disease later in life (8). Besides, neonatal mortality, preeclampsia, gestational hypertension (GH), gestational diabetes (GDM), and cesarean delivery also commonly occurred in overweight and obese pregnant women (5, 9–11).

GH is a common disease that seriously threatens the safety and health of pregnant women and their newborns (12). The incidence of this disease is about 10% worldwide (13). The higher incidence not only comes from the physical condition of pregnant women but also from the pressure of life, work, and family (14). Patients commonly experience symptoms such as hypertension, proteinuria, and edema (15). As the condition further deteriorates, patients may suffer from convulsions, coma, cerebral hemorrhage, heart failure, diffuse intravascular coagulation, and even death in severe cases (15, 16). In recent years, as the topic of hypertension in pregnancy has been discussed, the attention to this issue has gradually increased. The pathogenesis of GH is not fully established, so there is no complete cure for this disease, but preventative techniques are available (17, 18).

Exercise is widely recognized as a health maintenance method and it has numerous benefits (19, 20). It is acknowledged that exercise may accelerate metabolism, build cardiorespiratory capacity, improve immunity, and relieve mental stress (21–23). More notably, exercise can reduce complications in pregnant women by preventing weight gain or promoting weight loss (24, 25). However, the controversy of whether physical exercise (PE) may reduce the risk of GH in overweight and obese pregnant women always exists. In a previously published meta-analysis by Xing et al. (26), it was noted that exercise intervention reduced the risk of GH in overweight/obese pregnant women, while in the meta-analysis by Muhammad et al. (27) and meta-analysis by Du et al. (28), contrary conclusions were proposed. They found that PE was associated with a statistically non-significant decreased risk of GH. In the previous meta-analyses, the number of the included original studies was relatively small and subgroup analyses were not performed. Considering the serious consequences of GH, including the harm to maternal and infants, as well as the controversial conclusions from previous meta-analyses, it is of great value to conduct an updated meta-analysis to reassess this association.

## Methods

The Preferred Reporting Items declared by the Systematic Review and Meta-Analysis (PRISMA) was utilized to conduct this updated meta-analysis (29).

### Search strategy

The articles from inception to April 2022, presenting studies investigating exercise intervention and pregnancy outcomes were explored across several online databases, including Web of Science, Cochrane Library, Embase, PubMed, China Biomedical Database (CBM), VIP (Chinese) database, China National Knowledge Infrastructure, and Wanfang Data. The searching terms used were as follows: (exercise OR training OR activity OR exercise intervention) AND (pregnancy OR pregnant woman OR delivery) AND (essential hypertension OR hypertension OR hypertension in pregnancy OR gestational hypertension) AND (RCT OR randomized clinical trials OR randomized controlled clinical trial OR randomized controlled trial OR randomized controlled trials OR randomized experiment OR rct). The keywords used in the Chinese databases were replaced by the Chinese words with the same meaning as the English searching terms. For a comprehensive search to collect more qualified articles, studies were also collected through references from original published studies and relevant reviews. The records were processed with literature management software (EndNote, version 20) to exclude duplicates and to further screen the literature.

### Inclusion and exclusion criteria

Studies could be included in this the subsequent analysis if the following inclusion criteria were fulfilled: (1) the subjects of the study were pregnant women; (2) the pregnant women were overweight or obese (no specific definition as long as the original studies reported the participants were overweight or obese); (3) the intervention was exercise (no limitation on the type of exercise); (4) The outcome of GH should be reported (no specific definition as long as the original studies reported the participants have GH) (5) the study design was restricted to randomized controlled trial study. The exclusion criteria were also set: (1) Preclinical study; (2) observational studies; (3) reviews, case reports, meta-analysis, guidelines; (4) Duplicate articles; (5) Unable to extract data.

### Data extraction and quality assessment

Two authors (H. Tao and M. Liu) independently carried out the data extraction process using a pre-specified Excel form. Any dissonance was resolved with a senior supervisor (E. Xie) through discussion and consensus. Information extracted contents were listed as follows: (1) Basic information for the included articles (the first author's name, year of publication, geographic locations, the quality of the studies). (2) Baseline characteristics of the subjects in the eligible literature (sample size, ethnicity, pre-gestational BMI, gestational week, mothers' age). (3) Specific details of interventions (intervention measure and frequency). (4) The outcome indicators and outcome measures of interest (the event number in the experimental group, the total number in the experimental group, the event number in the control group, and the total number in the control group).

The quality of the included RCTs was assessed using the Jadad scoring scale for four indicators: literature random sequence generation, allocation concealment, blinding, and whether details of study participant withdrawal or dropout were described. Studies scoring 4–7 were considered of high quality and 1–3 were considered low-quality studies (30, 31).

### Statistical analysis

The Stata version 15.1 statistical software (Stata Corporation, College Station, TX) was utilized in this meta-analysis. Since GH is a dichotomous variable, the results were combined using the Mantel-Haenszel method and expressed as the pooled RR with the corresponding 95% confidence interval (CI). Heterogeneity among the included studies was estimated and tested by Q test and *I*^2^ statistic. Random-effects model would be applied if the test demonstrated a substantial level of heterogeneity (*I*^2^ > 50%), otherwise, a fixed-effects model would be applied (*I*^2^ < 50%) (32, 33). Subgroup analyses was performed according to geographic locations, sample size, exercise intervention measures and dietary interventions. Sensitive analysis was conducted to explore whether the result would depend on a particular study (34). Funnel plots, Egger's test, and Begg's test were used to estimate the potential for publication bias (35, 36). Statistical significance was set at *P*< *0.05*.

## Result

### Study selection and study characteristics

**Figure 1:**
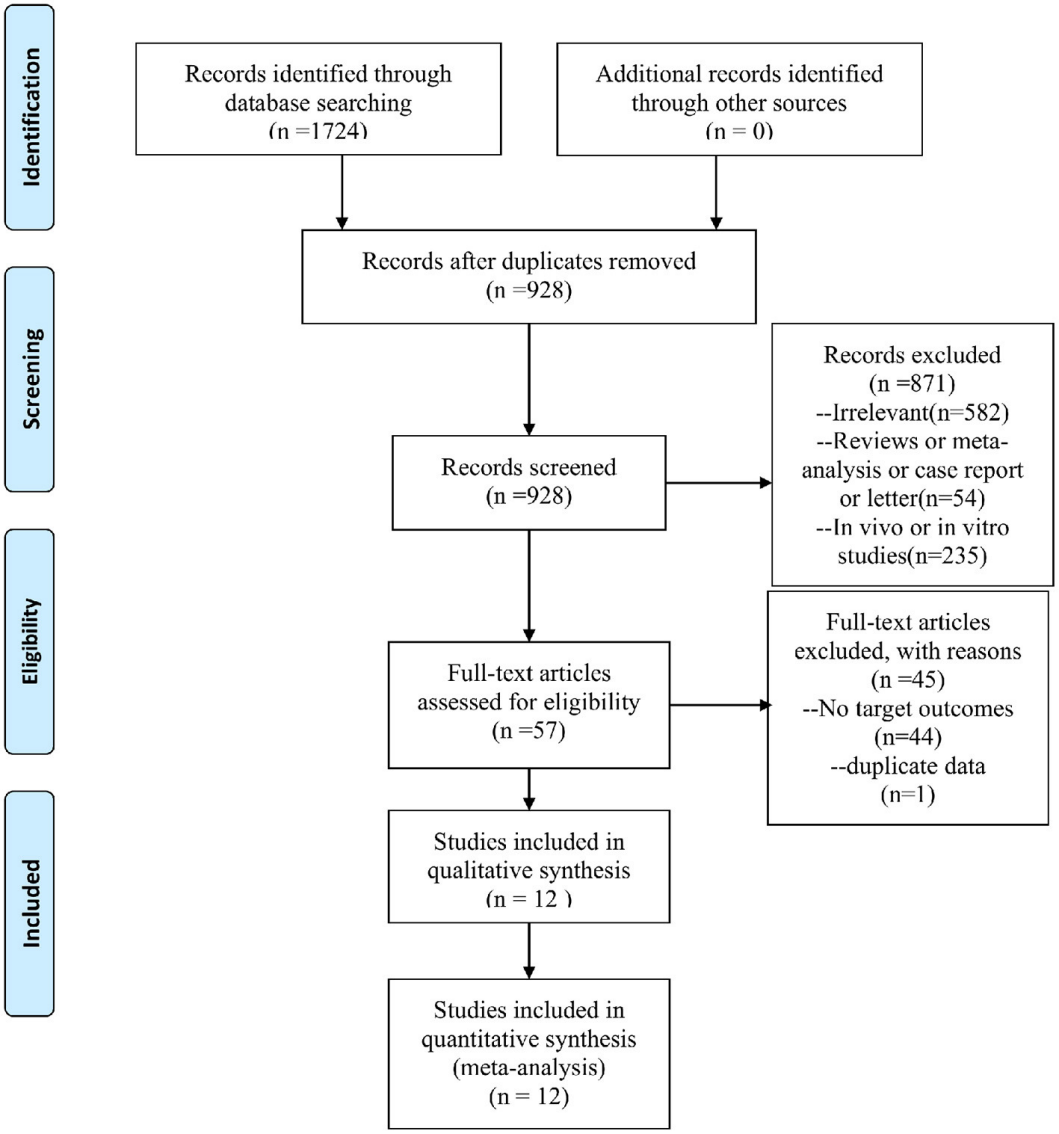
PRISMA flow chart.

Of the 1,724 potentially relevant documents initially searched (644 citations from PubMed, 473 citations from Embase, 32 citations from Cochrane Library, 204 citations from Web of Science, and 371 citations from all the Chinese databases), 928 remained after excluding duplicates. After subsequent scanning of the titles and abstracts, 871 articles were eliminated, mainly for the irrelevance to the aim of this meta-analysis and non-conformity of the study type. Full texts of the 57 records that remained were scrutinized. Finally, 12 articles (35–46) that met the criteria were selected. The flowchart of the searching and selecting process was presented in Figure 1.

Twelve documents involving maternal outcomes in pregnant women were included in this meta-analysis. These studies were published between 2014 and 2021. Of all, five were conducted in China, three were from Italy, one was from Norway, one was from Spanish, one was from Denmark and the rest one was from New Zealand. More detailed characteristics and information were summarized in Table 1.

**Table 1:** Characteristics of individual studies included in this meta-analysis.

**References**	**Country**	**Participants, n**	**Ethnicity**	**Pre-gestational BMI**	**Mothers' age** **(years**, **mean** **±SD)**		**Gestational** **week** **(weeks**, **mean** **±SD)**		**Intervention** **measures**		**Dietary** **interventions**		**Number of** **GH/total** **number**		**Quality**
					**Experimental group**	**Control group**	**Experimental group**	**Control group**	**Experimental group**	**Control group**	**Experimental group**	**Control group**	**Experimental group**	**Control group**	
Barakat et al. (37)	Spain	222	Caucasian	N/A	N/A	N/A	N/A	N/A	3 times/week, 50–55 min/session, involved aerobic exercise, aerobic dance, muscular strength, and flexibility	Visit with health care providers during pregnancy	No	No	6/107	7/115	5
Bruno et al. (38)	Italy	131	N/A	N/A	31.5 ± 5	30.8 ± 5.5	N/A	N/A	30 min of moderate intensity activity at least three times a week	Standard of Care	Low saturated fat diet with a total intake of 1,500 kcal/day	Hypocaloric, low-glycaemic, low-saturated fat diet	2/69	13/62	4
Ding et al. (39)	China	215	N/A	27.8 ± 2.7	30.6 ± 2.8	30.1 ± 2.7	8.6 ± 1.0	8.9 ± 1.4	Three face-to-face sessions about personalized dietary and exercise intervention, taking a walk for at least 6000 steps per day	A general advice session about weight management	50–60% carbohydrate, <30% fat, and 1.0–1.3 g/kg (IBW)/d protein	A general advice session about pregnancy nutrition	7/104	9/111	4
Fang and Li (40)	China	180	N/A	N/A	27.61 ± 1.88	27.24 ± 1.95	7.89 ± 1.32	7.96 ± 1.28	Walking: 15–25 min/day, 2 times/ week	Normal pregnancy test	Development of nutritional intervention programs based on individualized characteristics of pregnant women	Development of nutritional intervention programs based on individualized characteristics of pregnant women	5/90	7/90	5
Garnæs et al. (41)	Norway	74	N/A	33.9 ± 3.8	31.3 ± 3.8	31.4 ± 4.7	N/A	N/A	3 times weekly, 60 min and consisted of treadmill walking/jogging for 35 min (endurance training) and resistance training for large muscle groups and the pelvic floor muscles for 25 min	Ordinary maternity care	No	No	3/38	7/36	4
Huang (42)	China	120	N/A	N/A	25.6 ± 3.4	25.4 ± 3.5	10.2 ± 0.7	10.1 ± 0.8	30 min/day,5 times/week of low-intensity continuous aerobic exercise such as aerobics, walking, jogging, swimming, tai chi, etc. during pregnancy	Ordinary maternity care	Personalize the nutrition and energy requirements of pregnant women at different stages, and guide them to have a reasonable diet	Personalize the nutrition and energy requirements of pregnant women at different stages, and guide them to have a reasonable diet	3/60	10/60	5
Menichini et al. (43)	Italy	82	N/A	N/A	30.5 ± 4.4	30.4 ± 5.5	N/A	N/A	30 min/day walking at least 4 times/week	Normal pregnancy test	A low glycemic index, low saturated fat diet with a total intake of 1700 kcal/day	No	6/36	4/46	4
Petrella et al. (44)	Italy	61	N/A	N/A	31.5 ± 4.2	32.4 ± 5.9	12	12	Mild physical activity (30 min/day, 3 times/week)	Normal standard of Care	<1,700 kcal/day	<1,700 kcal/day	1/33	7/28	4
Renault et al. (45)	Denmark	259	N/A	N/A	30.9 ± 4.9	31.3 ± 4.2	N/A	N/A	Walking daily, aiming at a daily step count of 11,000	No	Hypocaloric low-fat diet with 1,200–1,675 kcal	Hypocaloric low-fat diet with 1,200–1,675 kcal	9/125	12/134	4
Seneviratne et al. (46)	New Zealand	74	N/A	N/A	N/A	N/A	N/A	N/A	Frequency varying between three and five sessions per week, and duration of moder ate-intensity exercise between 15 and 30 min per session, according to stage of pregnancy	No	No	No	1/37	0/37	4
Wang et al. (47)	China	100	N/A	N/A	N/A	N/A	N/A	N/A	Walking 9,000– 10,000 steps per day	Routine health education, regular blood glucose testing	Customized personalized diet plan	Customized personalized diet plan	3/50	4/50	4
Zhao et al. (48)	China	131	N/A	N/A	25 ± 7.6	25 ± 7.5	N/A	N/A	Walking: 30–60 min/day, 3 times/week; Pregnancy exercise, qigong, taijiquan, 3–5 times a week, 30 min each time	No	Individualized dietary interventions	Individualized dietary interventions	7/87	7/44	4

N/A, not applicable; GH, gestational hypertension.

### Overall meta-analysis

**Figure 2:**
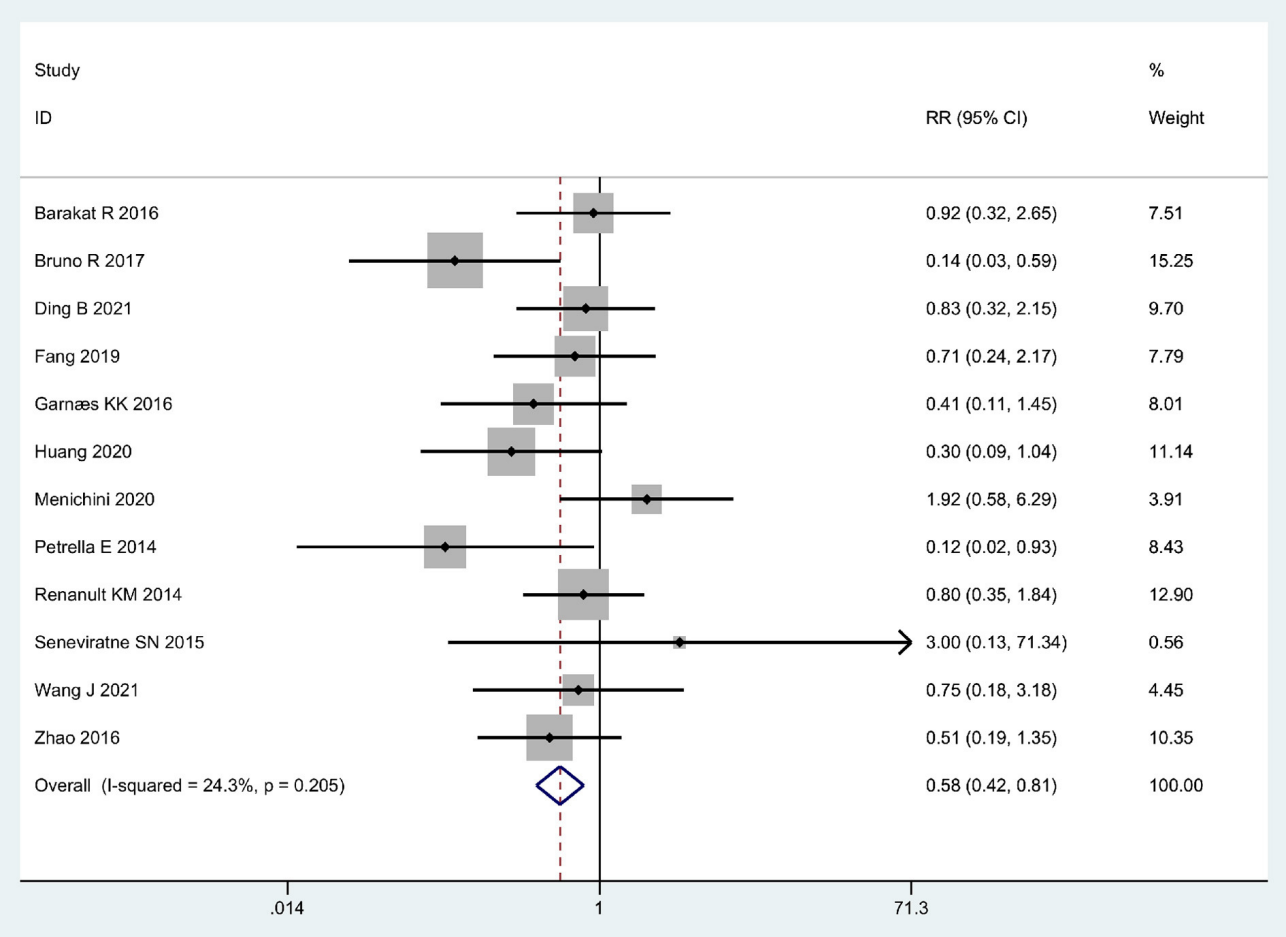
Forrest plot: Association between physical exercise and the risk of GH in obese and overweight pregnant women.

Twelve articles (37–48) regarding the relationship between exercise and GH were included in this meta-analysis. The result presented the RR through the fixed-effects model, which indicated that exercise was associated with a lower risk of GH in overweight or obese pregnant women (Pooled RR = 0.58, 95% CI = 0.42–0.81, *P* = 0.001; *I*^2^ = 24.3%; Figure 2).

### Subgroup analyses

In the subgroup analyses performed by geographic locations, a statistically significant association was observed in eastern countries (RR = 0.59, 95% CI = 0.36–0.96, *P* = *0.033*), while no statistically significant association was detected in Western countries (RR = 0.60, 95% CI = 0.29–1.26, *P* = *0.175*). In terms of sample size, the outcomes were statistically significant in both the groups with sample size > 120 and ≤120 group (RR = 0.60, 95% CI = 0.40–0.90, *P* = *0.014*; RR = 0.55, 95% CI = 0.32–0.95, *P* = *0.032*, respectively). For Exercise intervention measures, seven studies with intervention of mixed physical exercise indicated a significant impact of exercise on reducing the risk of developing GH in in obese and overweight pregnant women RR = 0.38, 95% CI = 0.24–0.62, *P* = *0.001*); however, in the five studies with intervention of walking only, the association between walking and incidence of GH was not statistically significant (RR = 0.90, 95% CI = 0.57–1.43, *P* = *0.650*). In case of dietary interventions, the pooled RR for combining dietary interventions with PE was 0.55, (95% CI = 0.39–0.79, *P* = 0.001), and the pooled RR for no dietary interventions was 0.74 (95% CI = 0.35–1.57, *P* = 0.403). All these results of subgroup analyses were represented in Table 2.

**Table 2:** Summary of pooled RRs with confidence interval (CI) in subgroups analyses.

**Analysis**	**No. of studies**	**RR (95%CI)**	**Heterogeneity**		**Significant**		**Model**
			***P***	**I^2^ (%)**	**Z**	***P***	
Overall	12	0.58 (0.42, 0.81)	0.205	24.3	3.26	0.001	Fixed
**Geographic locations**
Western countries	7	0.60 (0.29, 1.26)	0.050	52.4	1.36	0.175	Random
Eastern countries	5	0.59 (0.36, 0.96)	0.744	0	2.13	0.033	Fixed
**Sample size**
>120	6	0.60 (0.40, 0.90)	0.337	12.3	2.47	0.014	Fixed
≤120	6	0.55 (0.32, 0.95)	0.118	43.0	2.15	0.032	Fixed
**Exercise intervention measures**
Walking only	5	0.90 (0.57, 1.43)	0.757	0	0.45	0.650	Fixed
Mixed	7	0.38 (0.24–0.62)	0.250	23.5	3.96	0.001	Fixed
**Dietary interventions**	12						
Yes	9	0.55 (0.39–0.79)	0.123	36.9	3.23	0.001	Fixed
No	3	0.74 (0.35–1.57)	0.413	0	0.79	0.430	Fixed

### Sensitivity analyses and publication bias

**Figure 3:**
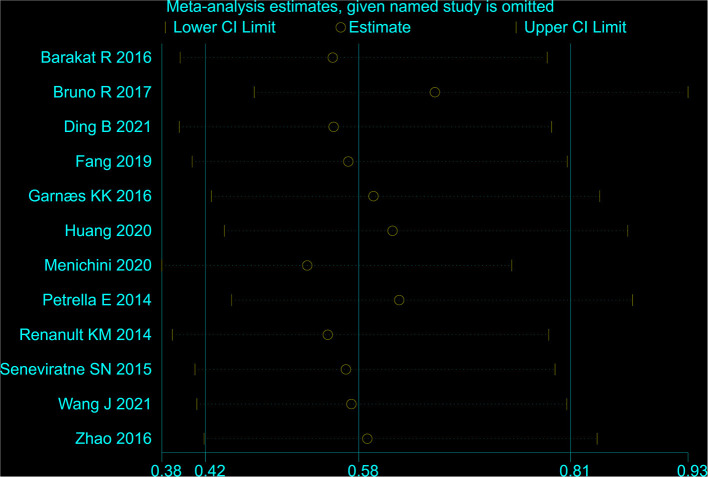
The result of the sensitivity analyses.

**Figure 4:**
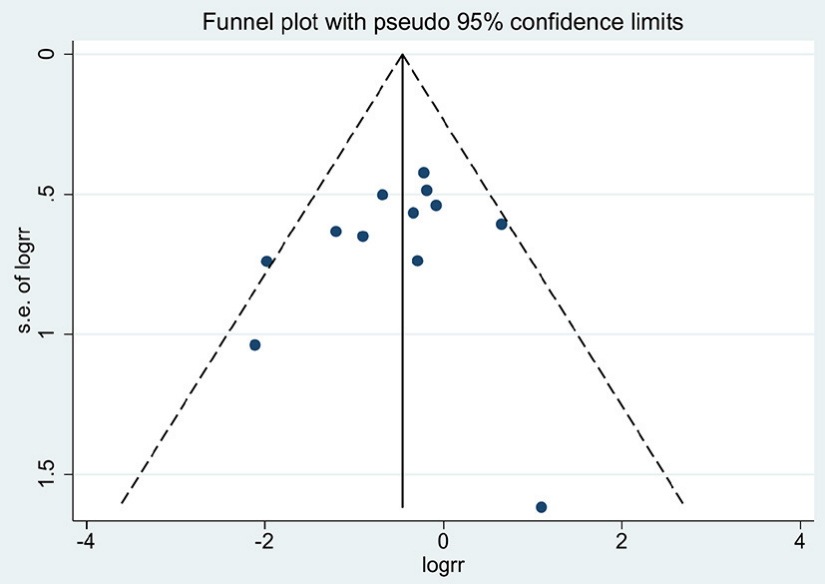
Funnel plot of this meta-analysis.

After leaving an individual study out at a time, the fluctuation of the pooled RRs was found to be between 0.53 and 0.66 with an upper limit of 95% CI constantly remaining <1, and *P*-value constantly remained <0.05, which indicated high stability of this meta-analysis (Figure 3). By changing the fixed-effect model to the random-effect model, the overall result was not altered significantly (Pooled RR = 0.62, 95% CI = 0.41–0.92), which further confirmed the stability. No apparent evidence of asymmetry was observed by visual inspection of the funnel plot (Figure 4). Begg's test (*Z* = *1.03*; *P* = *0.304*), Egger's test (*t* = –* 0.79; P* = *0.450*) for GH showed that publication bias might not exist.

## Discussion

Meta-analysis is considered an essential statistical tool to synthetically evaluate the effects of intervention for disease more precisely. Thus, we performed this updated meta-analysis, which identified a 0.58-fold decrease in the risk of developing GH in obese and overweight pregnant women. This finding was inconsistent with the previous meta-analysis by Muhammad et al. (27) and by Du et al. (28), they both found that exercise intervention would not reduce the risk of GH in overweight/obese pregnant women. However, the meta-analysis by Xing et al. (26) yielded a similar conclusion to our study. In the previous meta-analysis, the searching process may not have been comprehensive and therefore the results obtained do not seem to be very accurate. Comparing this study with the previous meta-analyses (26–28), in this study, we conducted a more detailed and comprehensive search, included more articles, had a larger sample size, and therefore had greater statistical validity and more feasible results.

Besides, subgroup analysis confirmed the relevance between exercise and the decreased risk of GH, because the outcomes were statistically significant in both the groups with sample size > 120 and ≤120 groups. In terms of the geographic locations, a positive association was observed in eastern countries while studies in Western countries showed no such association. This might be explained by discrepancies in different ethnicities between Asian and Western countries. The different lifestyles and dietary habits of different regions cause differences in the characteristics of the general population, and therefore the characteristics of pregnant women, differ significantly from one another. A meta-analysis (49) using the 2009 IOM guidelines in the global population indicated that the highest mean gestational weight gain (GWG) and pre-pregnancy BMI were in Western countries and the lowest were in Eastern countries. It is assumed that a higher BMI before pregnancy and a higher GWG during pregnancy may also be a risk factors for GH. However, due to the limitation of the number of articles, a stratified analysis based on pre-pregnancy BMI was not possible. In the case of the exercise pattern, a statistically significant reduction of 62% was observed in mixed exercise style, whereas no significant association was detected in walking only. Such results suggest that pregnant women should be exposed to different types of moderate-intensity exercise during pregnancy than walking only. It is recommended that pregnant women can combine walking, jogging, yoga, and tai chi to reduce the incidence of GH. More specific sports intensities and durations need to be explored in more depth. In case of dietary interventions, we found that combining dietary interventions with PE had better effect for pregnant women, which suggested that dietary interventions are as important as exercise interventions in the daily care of overweight or obese pregnant women. Dietary interventions can change the structure of pregnant women's diet by reducing the fat content and glucose load of the diet and increasing the protein and fiber content. These low glycemic index diets reduce weight gain during pregnancy and also reduce the risk of GH (50).

The exercise of pregnant women is greatly reduced and the intake of food is increased, so there are a lot of calories in the body that cannot be consumed, which is easy to lead to fat accumulation and obesity symptoms. These pregnant women are prone to GH due to abnormal lipid metabolism (51). It is known that adipose tissue is involved in metabolic syndromes such as obesity and hyperlipidemia, which can result in inflammatory changes that then cause increased oxidative stress. This may lead to endothelial dysfunction, and ultimately to clinical disorders such as GH (52). In clinical, many pregnant women are not aware of the harmful effects of obesity, which requires clinicians to strengthen guidance to control the BMI of obese pregnant women, of which exercise intervention is the simplest and most important measure. In an observational study by Hutcheon et al., the reasonable range of weight control was reported. In normal-weight women, lowest risk of adverse perinatal outcome was observed at a weight-gain z score of 20.2 SDs. With a non-inferiority margin of 20%, risks of adverse outcome were not meaningfully increased from the 20.2-SD reference value between z scores of 20.97 and +0.33 SDs (which corresponded to 11.3–18.4 kg). In overweight women, the recommended range was much broader: 22.11 to +0.29 SDs (4.4–18.1 kg) (53). Individualized exercise instruction can be performed for pregnant women, and appropriate exercise programs can be developed based on their exercise status and changes in body mass at different times during pregnancy.

Several inherent limitations need to be cited when interpreting the results of this meta-analysis. First, the exercise duration and intensity were different across the included studies, and due to the limited number of the included studies, results cannot be stratified by these factors. Third, the majority of studies were conducted in Europe and Asia, with limited research in other regions. Last, it is not possible to conduct a stratified analysis based on pre-pregnancy BMI. Despite the limitations, the strength of this article should be highlighted. To begin with, compared with the previous meta-analysis, more studies were enrolled. The large sample size provided stronger statistical power. Second, more comprehensive subgroup analyses were performed in this study. Third, no obvious publication bias was detected.

## Conclusion

In conclusion, exercise is associated with a reduced risk of GH in overweight and/or obese pregnant women. Exercise is a convenient intervention in general life. Given the prevalence of GH is increasing, the effect of general exercise on GH in our study is promising. However, more well-designed studies are warranted to further elaborate on these associations.

## Data availability statement

The original contributions presented in the study are included in the article/Supplementary material, further inquiries can be directed to the corresponding author/s.

## Author contributions

EX, CL, and QZ conceived and designed the research, provided critical opinions, and revised the manuscript. HT and ML designed the research, performed the statistical analysis, interpret data, and wrote the manuscript. All authors approved the final manuscript.

## Conflict of interest

The authors declare that the research was conducted in the absence of any commercial or financial relationships that could be construed as a potential conflict of interest.

## Publisher's note

All claims expressed in this article are solely those of the authors and do not necessarily represent those of their affiliated organizations, or those of the publisher, the editors and the reviewers. Any product that may be evaluated in this article, or claim that may be made by its manufacturer, is not guaranteed or endorsed by the publisher.
