# Supplementary material for: The effect of exercise on the prevention of gestational hypertension in obese and overweight pregnant women: An updated meta-analysis
Source: Front Public Health. 2022 Aug 15;10:923161. doi: 10.3389/fpubh.2022.923161 (PMC9420989; doi:10.3389/fpubh.2022.923161)
Supplement: Supplementary file 1 [file Data_Sheet_1.docx]

**PubMed**

(exercise OR training OR activity OR exercise intervention) AND (pregnancy OR pregnant woman OR delivery) AND (essential hypertension OR hypertension OR hypertension in pregnancy OR gestational hypertension) AND (RCT OR randomized clinical trials OR randomized controlled clinical trial OR randomized controlled trial OR randomized controlled trials OR randomized experiment OR rct)

**China National Knowledge Infrastructure（中国知网）**

（运动 + 训练 + 锻炼）AND （怀孕 + 孕妇）AND （高血压）AND （随机 + 随机对照 + RCT）

**VIP (Chinese) database （维普数据库）**

（运动 OR 训练 OR 锻炼）AND （怀孕 OR 孕妇）AND （高血压）AND （随机 OR 随机对照 OR RCT）

**Wanfang Data （万方数据库）**

（运动 或 训练 或 锻炼）与 （怀孕 或 孕妇）与 （高血压）与 （随机 或 随机对照 或 RCT）

**China Biomedical Database (CBM数据库)**

（运动 OR 训练 OR 锻炼）AND （怀孕 OR 孕妇）AND （高血压）AND （随机 OR 随机对照 OR RCT）

**Embase**

(exercise OR training OR activity OR exercise intervention) AND (pregnancy OR pregnant woman OR delivery) AND (essential hypertension OR hypertension OR hypertension in pregnancy OR gestational hypertension) AND (RCT OR randomized clinical trials OR randomized controlled clinical trial OR randomized controlled trial OR randomized controlled trials OR randomized experiment OR rct)

**Cochrane Library**

(exercise OR training OR activity OR exercise intervention) AND (pregnancy OR pregnant woman OR delivery) AND (essential hypertension OR hypertension OR hypertension in pregnancy OR gestational hypertension) AND (RCT OR randomized clinical trials OR randomized controlled clinical trial OR randomized controlled trial OR randomized controlled trials OR randomized experiment OR rct)

**Web of science**

(exercise OR training OR activity OR exercise intervention) AND (pregnancy OR pregnant woman OR delivery) AND (essential hypertension OR hypertension OR hypertension in pregnancy OR gestational hypertension) AND (RCT OR randomized clinical trials OR randomized controlled clinical trial OR randomized controlled trial OR randomized controlled trials OR randomized experiment OR rct)
